# Methyl 2-(5-methyl-3-methyl­sulfinyl-1-benzofuran-2-yl)acetate

**DOI:** 10.1107/S1600536808024689

**Published:** 2008-08-06

**Authors:** Hong Dae Choi, Pil Ja Seo, Byeng Wha Son, Uk Lee

**Affiliations:** a Department of Chemistry, Dongeui University, San 24 Kaya-dong, Busanjin-gu, Busan 614-714, Republic of Korea; b Department of Chemistry, Pukyong National University, 599-1 Daeyeon 3-dong, Nam-gu, Busan 608-737, Republic of Korea

## Abstract

The title compound, C_13_H_14_O_4_S, was prepared by oxidation of methyl 2-(5-methyl-3-methyl­sulfanyl-1-benzofuran-2-yl)acetate with 3-chloro­peroxy­benzoic acid. The O atom and methyl group of the methyl­sulfinyl substituent lie on opposite sides of the plane of the benzofuran system. The crystal structure is stabilized by inter­molecular aromatic π–π inter­actions between the benzene rings of neighbouring mol­ecules, with a centroid–centroid separation of 3.841 (3) Å.

## Related literature

For the crystal structures of similar ethyl 2-(3-methyl­sulfinyl-1-benzofuran-2-yl)acetate derivatives, see: Choi *et al.* (2007*a* ▶,*b* ▶).
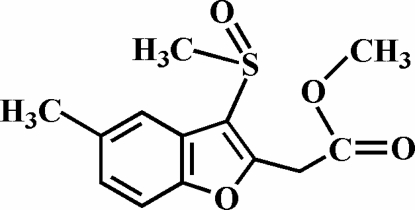


## Experimental

### 

#### Crystal data

C_13_H_14_O_4_S
*M* *_r_* = 266.30
Triclinic, 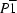

*a* = 7.9331 (6) Å
*b* = 8.1097 (6) Å
*c* = 10.7017 (8) Å
α = 71.601 (1)°
β = 81.107 (1)°
γ = 84.303 (1)°
*V* = 644.51 (8) Å^3^
*Z* = 2
Mo *K*α radiation
μ = 0.26 mm^−1^
*T* = 298 (2) K
0.40 × 0.20 × 0.20 mm

#### Data collection

Bruker SMART CCD diffractometer
Absorption correction: none
3414 measured reflections
2237 independent reflections
1788 reflections with *I* > 2σ(*I*)
*R*_int_ = 0.052

#### Refinement

*R*[*F* ^2^ > 2σ(*F* ^2^)] = 0.045
*wR*(*F* ^2^) = 0.123
*S* = 1.03
2237 reflections
166 parameters
H-atom parameters constrained
Δρ_max_ = 0.34 e Å^−3^
Δρ_min_ = −0.35 e Å^−3^

### 

Data collection: *SMART* (Bruker, 2001 ▶); cell refinement: *SAINT* (Bruker, 2001 ▶); data reduction: *SAINT*; program(s) used to solve structure: *SHELXS97* (Sheldrick, 2008 ▶); program(s) used to refine structure: *SHELXL97* (Sheldrick, 2008 ▶); molecular graphics: *ORTEP-3* (Farrugia, 1997 ▶) and *DIAMOND* (Brandenburg, 1998 ▶); software used to prepare material for publication: *SHELXL97*.

## Supplementary Material

Crystal structure: contains datablocks global, I. DOI: 10.1107/S1600536808024689/bh2186sup1.cif

Structure factors: contains datablocks I. DOI: 10.1107/S1600536808024689/bh2186Isup2.hkl

Additional supplementary materials:  crystallographic information; 3D view; checkCIF report

## Figures and Tables

**Fig. 1.**
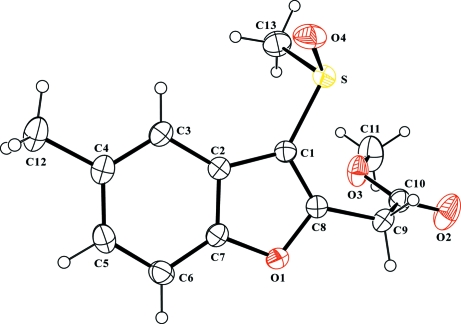
The molecular structure of the title compound, showing displacement ellipsoids drawn at the 30% probability level.

**Fig. 2.**
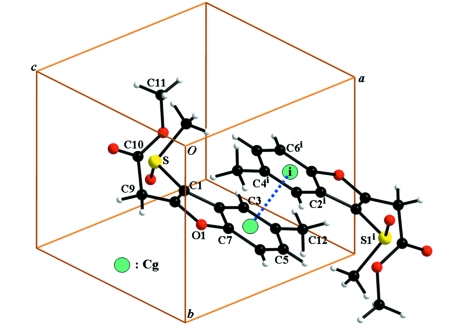
Intermolecular π–π interactions (dotted lines) in the title compound. Cg denotes ring centroid. Symmetry code: (i) 1-x, 2-y, -z.

## supplementary crystallographic information

### Comment

This work is related to our previous communications on the synthesis and structures of ethyl 2-(3-methylsulfinyl-1-benzofuran-2-yl)acetate analogues, *viz*. ethyl 2-(5-chloro-3-methylsulfinyl-1-benzofuran-2-yl)acetate (Choi *et al.*, 2007*a*) and ethyl 2-(5-methyl-3-methylsulfinyl-1-benzofuran-2-yl)acetate (Choi *et al.*, 2007*b*). Here we report the crystal structure of the title compound, methyl 2-(5-methyl-3-methylsulfinyl-1-benzofuran-2-yl)acetate (Fig. 1).

The benzofuran unit is essentially planar, with a mean deviation of 0.009 (2) Å from the least-squares plane defined by the nine constituent atoms. The packing structure is stabilized by aromatic π–π stacking interactions between adjacent benzene units, with a *Cg*···*Cg*^i^ distance is 3.841 (3) Å (Fig. 2).

### Experimental

77% 3-Chloroperoxybenzoic acid (359 mg, 1.6 mmol) was added in small portions to a stirred solution of methyl 2-(5-methyl-3-methylsulfanyl-1-benzofuran-2-yl)acetate (375 mg, 1.5 mmol) in dichloromethane (30 ml) at 273 K. After being stirred for 3 h at room temperature, the mixture was washed with saturated sodium bicarbonate solution and the organic layer was separated, dried over magnesium sulfate, filtered and concentrated under vacuum. The residue was purified by column chromatography (ethyl acetate) to afford the title compound as a colorless solid [yield 79%, m.p. 380–381 K; *R*_f_ = 0.58 (ethyl acetate)]. Single crystals suitable for X-ray diffraction were prepared by evaporation of a solution of the title compound in ethyl acetate at room temperature. Spectroscopic analysis: ^1^H NMR (CDCl_3_, 400 MHz) δ 2.45 (s, 3H), 3.07 (s, 3H), 3.74 (s, 3H), 4.04 (s, 2H), 7.17 (dd, *J* = 8.44 Hz and *J* = 1.08 Hz, 1H), 7.38 (d, *J* = 8.40 Hz, 1H), 7.71 (s, 1H); EI–MS 266 [*M*^+^].

### Refinement

All H atoms were geometrically positioned and refined using a riding model, with C—H = 0.93 Å for aromatic H atoms, 0.96 Å for methyl H atoms and 0.97 Å for methylene H atoms, respectively, and with *U*_iso_(H) = 1.2Ueq(C) for aromatic and methylene H atoms and 1.5Ueq(C) for methyl H atoms.

### Crystal data

**Table:**

C_13_H_14_O_4_S	*Z* = 2
*M**_r_* = 266.30	*F*_000_ = 280
Triclinic, *P*1	*D*_x_ = 1.372 Mg m^−^^3^
Hall symbol: -P 1	Melting point = 380–381 K
*a* = 7.9331 (6) Å	Mo *K*α radiation λ = 0.71073 Å
*b* = 8.1097 (6) Å	Cell parameters from 1817 reflections
*c* = 10.7017 (8) Å	θ = 2.6–27.3º
α = 71.601 (1)º	µ = 0.26 mm^−^^1^
β = 81.107 (1)º	*T* = 298 (2) K
γ = 84.303 (1)º	Block, colorless
*V* = 644.51 (8) Å^3^	0.40 × 0.20 × 0.20 mm

### Data collection

**Table:**

Bruker SMART CCD diffractometer	2237 independent reflections
Radiation source: fine-focus sealed tube	1788 reflections with *I* > 2σ(*I*)
Monochromator: graphite	*R*_int_ = 0.052
Detector resolution: 10.0 pixels mm^-1^	θ_max_ = 25.0º
*T* = 298(2) K	θ_min_ = 2.0º
φ and ω scans	*h* = −9→9
Absorption correction: none	*k* = −9→9
3414 measured reflections	*l* = −9→12

### Refinement

**Table:**

Refinement on *F*^2^	Secondary atom site location: difference Fourier map
Least-squares matrix: full	Hydrogen site location: inferred from neighbouring sites
*R*[*F*^2^ > 2σ(*F*^2^)] = 0.045	H-atom parameters constrained
*wR*(*F*^2^) = 0.123	*w* = 1/[σ^2^(*F*_o_^2^) + (0.0647*P*)^2^ + 0.1924*P*] where *P* = (*F*_o_^2^ + 2*F*_c_^2^)/3
*S* = 1.04	(Δ/σ)_max_ < 0.001
2237 reflections	Δρ_max_ = 0.34 e Å^−^^3^
166 parameters	Δρ_min_ = −0.35 e Å^−^^3^
Primary atom site location: structure-invariant direct methods	Extinction correction: none

### Fractional atomic coordinates and isotropic or equivalent isotropic displacement parameters (Å^2^)

**Table:**

	*x*	*y*	*z*	*U*_iso_*/*U*_eq_	
S	0.22227 (8)	0.36975 (8)	0.45695 (6)	0.0430 (2)	
O1	0.16435 (19)	0.5432 (2)	0.07927 (16)	0.0392 (4)	
O2	−0.1560 (3)	0.0853 (3)	0.3071 (3)	0.0850 (8)	
O3	0.1251 (2)	0.0969 (2)	0.2923 (2)	0.0565 (5)	
O4	0.2489 (3)	0.5059 (3)	0.51807 (19)	0.0597 (5)	
C1	0.2418 (3)	0.4676 (3)	0.2838 (2)	0.0348 (5)	
C2	0.3713 (3)	0.5757 (3)	0.1944 (2)	0.0342 (5)	
C3	0.5246 (3)	0.6396 (3)	0.2059 (3)	0.0410 (6)	
H3	0.5647	0.6111	0.2877	0.049*	
C4	0.6143 (3)	0.7457 (3)	0.0930 (3)	0.0445 (6)	
C5	0.5523 (3)	0.7870 (3)	−0.0291 (3)	0.0486 (7)	
H5	0.6147	0.8592	−0.1038	0.058*	
C6	0.4028 (3)	0.7256 (3)	−0.0442 (3)	0.0460 (6)	
H6	0.3628	0.7534	−0.1260	0.055*	
C7	0.3168 (3)	0.6199 (3)	0.0709 (2)	0.0366 (5)	
C8	0.1227 (3)	0.4533 (3)	0.2103 (2)	0.0352 (5)	
C9	−0.0416 (3)	0.3635 (3)	0.2452 (3)	0.0404 (6)	
H9A	−0.1049	0.4071	0.1697	0.048*	
H9B	−0.1081	0.3975	0.3181	0.048*	
C10	−0.0307 (3)	0.1679 (3)	0.2841 (3)	0.0447 (6)	
C11	0.1449 (4)	−0.0918 (4)	0.3298 (4)	0.0801 (11)	
H11A	0.0761	−0.1409	0.4132	0.120*	
H11B	0.2627	−0.1280	0.3382	0.120*	
H11C	0.1090	−0.1312	0.2629	0.120*	
C12	0.7804 (3)	0.8174 (4)	0.1000 (3)	0.0616 (8)	
H12A	0.8119	0.7677	0.1878	0.092*	
H12B	0.7661	0.9416	0.0797	0.092*	
H12C	0.8685	0.7884	0.0369	0.092*	
C13	0.4142 (4)	0.2333 (4)	0.4661 (3)	0.0573 (7)	
H13A	0.5112	0.3042	0.4396	0.086*	
H13B	0.4176	0.1635	0.4080	0.086*	
H13C	0.4167	0.1588	0.5556	0.086*	

### Atomic displacement parameters (Å^2^)

**Table:**

	*U*^11^	*U*^22^	*U*^33^	*U*^12^	*U*^13^	*U*^23^
S	0.0403 (4)	0.0487 (4)	0.0362 (4)	−0.0034 (3)	−0.0055 (3)	−0.0072 (3)
O1	0.0378 (9)	0.0428 (9)	0.0376 (9)	−0.0039 (7)	−0.0099 (7)	−0.0104 (7)
O2	0.0557 (13)	0.0589 (13)	0.135 (2)	−0.0209 (11)	−0.0173 (13)	−0.0150 (14)
O3	0.0415 (10)	0.0357 (9)	0.0832 (14)	−0.0039 (8)	0.0035 (9)	−0.0100 (9)
O4	0.0708 (13)	0.0711 (13)	0.0456 (11)	0.0066 (10)	−0.0141 (10)	−0.0297 (10)
C1	0.0363 (12)	0.0338 (12)	0.0349 (12)	−0.0017 (9)	−0.0056 (10)	−0.0110 (10)
C2	0.0354 (12)	0.0307 (11)	0.0377 (13)	0.0006 (9)	−0.0047 (10)	−0.0128 (10)
C3	0.0376 (13)	0.0413 (13)	0.0482 (15)	−0.0020 (10)	−0.0082 (11)	−0.0182 (12)
C4	0.0369 (13)	0.0363 (13)	0.0611 (17)	−0.0010 (10)	−0.0039 (12)	−0.0175 (12)
C5	0.0433 (14)	0.0415 (14)	0.0517 (16)	−0.0044 (11)	0.0054 (12)	−0.0060 (12)
C6	0.0492 (15)	0.0442 (14)	0.0397 (14)	−0.0006 (12)	−0.0058 (12)	−0.0065 (11)
C7	0.0337 (12)	0.0341 (12)	0.0435 (14)	0.0006 (9)	−0.0065 (10)	−0.0139 (10)
C8	0.0354 (12)	0.0317 (11)	0.0381 (13)	−0.0004 (9)	−0.0038 (10)	−0.0110 (10)
C9	0.0317 (12)	0.0448 (14)	0.0466 (15)	−0.0027 (10)	−0.0082 (11)	−0.0152 (11)
C10	0.0423 (14)	0.0478 (15)	0.0453 (15)	−0.0094 (11)	−0.0040 (11)	−0.0148 (12)
C11	0.070 (2)	0.0385 (16)	0.115 (3)	−0.0043 (15)	0.006 (2)	−0.0086 (17)
C12	0.0427 (15)	0.0532 (16)	0.088 (2)	−0.0115 (12)	−0.0069 (15)	−0.0182 (16)
C13	0.0571 (17)	0.0524 (16)	0.0596 (18)	0.0078 (13)	−0.0169 (14)	−0.0116 (14)

### Geometric parameters (Å, °)

**Table:**

S—O4	1.495 (2)	C5—H5	0.9300
S—C1	1.759 (2)	C6—C7	1.381 (3)
S—C13	1.788 (3)	C6—H6	0.9300
O1—C8	1.366 (3)	C8—C9	1.495 (3)
O1—C7	1.392 (3)	C9—C10	1.505 (3)
O2—C10	1.204 (3)	C9—H9A	0.9700
O3—C10	1.315 (3)	C9—H9B	0.9700
O3—C11	1.453 (3)	C11—H11A	0.9600
C1—C8	1.354 (3)	C11—H11B	0.9600
C1—C2	1.444 (3)	C11—H11C	0.9600
C2—C7	1.382 (3)	C12—H12A	0.9600
C2—C3	1.405 (3)	C12—H12B	0.9600
C3—C4	1.381 (4)	C12—H12C	0.9600
C3—H3	0.9300	C13—H13A	0.9600
C4—C5	1.397 (4)	C13—H13B	0.9600
C4—C12	1.513 (3)	C13—H13C	0.9600
C5—C6	1.382 (4)		
			
O4—S—C1	107.63 (11)	O1—C8—C9	116.0 (2)
O4—S—C13	105.85 (13)	C8—C9—C10	117.4 (2)
C1—S—C13	98.82 (13)	C8—C9—H9A	108.0
C8—O1—C7	106.00 (17)	C10—C9—H9A	108.0
C10—O3—C11	117.3 (2)	C8—C9—H9B	108.0
C8—C1—C2	107.3 (2)	C10—C9—H9B	108.0
C8—C1—S	122.09 (18)	H9A—C9—H9B	107.2
C2—C1—S	130.59 (18)	O2—C10—O3	123.6 (2)
C7—C2—C3	119.0 (2)	O2—C10—C9	121.9 (2)
C7—C2—C1	104.91 (19)	O3—C10—C9	114.4 (2)
C3—C2—C1	136.1 (2)	O3—C11—H11A	109.5
C4—C3—C2	118.5 (2)	O3—C11—H11B	109.5
C4—C3—H3	120.8	H11A—C11—H11B	109.5
C2—C3—H3	120.8	O3—C11—H11C	109.5
C3—C4—C5	120.0 (2)	H11A—C11—H11C	109.5
C3—C4—C12	120.5 (3)	H11B—C11—H11C	109.5
C5—C4—C12	119.4 (2)	C4—C12—H12A	109.5
C6—C5—C4	123.0 (2)	C4—C12—H12B	109.5
C6—C5—H5	118.5	H12A—C12—H12B	109.5
C4—C5—H5	118.5	C4—C12—H12C	109.5
C7—C6—C5	115.3 (2)	H12A—C12—H12C	109.5
C7—C6—H6	122.4	H12B—C12—H12C	109.5
C5—C6—H6	122.4	S—C13—H13A	109.5
C6—C7—C2	124.2 (2)	S—C13—H13B	109.5
C6—C7—O1	125.2 (2)	H13A—C13—H13B	109.5
C2—C7—O1	110.58 (19)	S—C13—H13C	109.5
C1—C8—O1	111.21 (19)	H13A—C13—H13C	109.5
C1—C8—C9	132.8 (2)	H13B—C13—H13C	109.5
			
O4—S—C1—C8	−129.6 (2)	C1—C2—C7—C6	179.0 (2)
C13—S—C1—C8	120.6 (2)	C3—C2—C7—O1	179.36 (19)
O4—S—C1—C2	48.5 (2)	C1—C2—C7—O1	−1.1 (2)
C13—S—C1—C2	−61.4 (2)	C8—O1—C7—C6	−178.8 (2)
C8—C1—C2—C7	0.5 (2)	C8—O1—C7—C2	1.2 (2)
S—C1—C2—C7	−177.78 (18)	C2—C1—C8—O1	0.3 (3)
C8—C1—C2—C3	180.0 (3)	S—C1—C8—O1	178.70 (15)
S—C1—C2—C3	1.7 (4)	C2—C1—C8—C9	−178.3 (2)
C7—C2—C3—C4	0.5 (3)	S—C1—C8—C9	0.1 (4)
C1—C2—C3—C4	−178.9 (2)	C7—O1—C8—C1	−0.9 (2)
C2—C3—C4—C5	−0.1 (4)	C7—O1—C8—C9	177.97 (19)
C2—C3—C4—C12	−179.8 (2)	C1—C8—C9—C10	−72.1 (3)
C3—C4—C5—C6	−0.3 (4)	O1—C8—C9—C10	109.3 (2)
C12—C4—C5—C6	179.4 (2)	C11—O3—C10—O2	0.7 (4)
C4—C5—C6—C7	0.3 (4)	C11—O3—C10—C9	179.8 (3)
C5—C6—C7—C2	0.2 (4)	C8—C9—C10—O2	−176.1 (3)
C5—C6—C7—O1	−179.8 (2)	C8—C9—C10—O3	4.8 (3)
C3—C2—C7—C6	−0.6 (3)		

## References

[bb1] Brandenburg, K. (1998). *DIAMOND* Crystal Impact GbR, Bonn, Germany.

[bb2] Bruker (2001). *SAINT* and *SMART* Bruker AXS Inc., Madison, Wisconsin, USA.

[bb3] Choi, H. D., Seo, P. J., Son, B. W. & Lee, U. (2007*a*). *Acta Cryst.* E**63**, o3832.

[bb4] Choi, H. D., Seo, P. J., Son, B. W. & Lee, U. (2007*b*). *Acta Cryst.* E**63**, o3839.

[bb5] Farrugia, L. J. (1997). *J. Appl. Cryst.* **30**, 565.

[bb6] Sheldrick, G. M. (2008). *Acta Cryst.* A**64**, 112–122. 10.1107/S0108767307043930 18156677

